# Low GAS5 expression may predict poor survival and cisplatin resistance in cervical cancer

**DOI:** 10.1038/s41419-020-2735-2

**Published:** 2020-07-13

**Authors:** Xingyu Fang, Guanglei Zhong, Yuhan Wang, Zhongqiu Lin, Rongchun Lin, Tingting Yao

**Affiliations:** 1 Department of Gynecological Oncology, Sun Yat-sen Memorial Hospital, Sun Yat-sen University, 107 Yan Jiang West Road, 510120 Guangzhou, People’s Republic of China; 2 Guangdong Provincial Key Laboratory of Malignant Tumor Epigenetics and Gene Regulation, Sun Yat-Sen Memorial Hospital, Sun Yat-Sen University, 510120 Guangzhou, China

**Keywords:** Oncogenes, Long non-coding RNAs

## Abstract

Cisplatin resistance is a major challenge in cervical cancer (CC) chemotherapy. Growth arrest‐specific 5 (GAS5) has been reported to be a tumour suppressor gene in CC. However, the mechanism of GAS5 in chemoresistance remains undetermined. Our research evaluated GAS5 expression in normal and CC tissues by qPCR and in situ hybridization (ISH). Statistical analysis was conducted to analyse the association of GAS5 expression with survival. Biochemical methods were used to screen upstream and downstream regulators of GAS5. Then, interactions were confirmed by ChIP, RNA pull-down, RNA immunoprecipitation (RIP), dual-luciferase reporter and real-time PCR assays. The cisplatin sensitivity of GAS5-overexpressing CC cells was demonstrated in vitro and in vivo. The results showed that low GAS5 expression was correlated with poor overall survival. Mechanistically, GAS5 was transcriptionally modulated by P-STAT3 and served as a competing endogenous RNA (ceRNA) of miR-21 to indirectly affect cisplatin sensitivity through PDCD4 regulation in CC cells. Animal studies confirmed that GAS5 enhanced cisplatin sensitivity and promoted PDCD4 expression in vivo. GAS5 was regulated by P-STAT3 and affected the sensitivity of CC to cisplatin-based chemotherapy through the miR-21/PDCD4 axis. This result may provide new insight into cisplatin-based therapy.

## Introduction

Cervical cancer (CC) remains the second leading cause of cancer death in women aged 20–39 years. CC mortality among women in less developed countries is twice that of women in affluent countries^1^. Persistent human papillomavirus (HPV) infection, most commonly with HPV16, is a high-risk factor for CC^2^. Despite advances in surgical resection, chemoradiotherapy and anti-angiogenic therapy for CC, the long-term survival rate of CC patients remains unsatisfactory due to metastasis and recurrence^3–5^. Currently, cisplatin (DDP)-based concurrent chemoradiotherapy is the standard treatment for locally advanced CC^6^. A study found that consolidation chemotherapy after cisplatin-based chemoradiotherapy can control distant relapse and improve overall survival (OS) and progression-free survival^7^. However, cisplatin resistance remains a serious problem during the treatment of CC, which greatly restricts the clinical application and efficacy of cisplatin^8^. Thus, investigating the theory and mechanism underlying the chemosensitivity of CC cells is an urgent task.

Chemotherapeutic resistance in CC is usually caused by changes in cellular drug uptake, decreased drug influx and increased drug efflux, drug detoxification via cellular mercaptans, drug target alterations and DNA repair^9^. In addition, hypoxia, epithelial–mesenchymal transition, apoptosis inhibition and changes in molecular signalling pathways also contribute to drug resistance^10^. Studies have suggested that cisplatin resistance is related to regulation of the cell cycle, apoptosis and the Wnt pathway by long non-coding RNAs (lncRNAs)^11^. LncRNAs are small non-coding RNAs with more than 200 nucleotides and account for 80% of all non-coding RNAs. LncRNAs can regulate gene transcription by binding with chromatin regulatory proteins and via gene silencing, DNA methylation, histone modification, hybridization and other mechanisms^12^. Growth arrest‐specific 5 (GaS5), located on chromosome 1q25, plays an anticancer role in breast, prostate and ovarian cancers and in other tumours^13–15^. Our previous studies have proven that GAS5 inhibits the proliferation, invasion, migration and apoptosis of CC cells^16^. However, the functional mechanism and the upstream transcription factor for GAS5 in CC remain unknown.

Recently, a large number of studies have suggested cross-modulation between lncRNAs and microRNAs (miRNAs). LncRNAs bind and sequester miRNAs and block their functions^17^. MiRNAs regulate gene expression by binding to the 3′-untranslated regions (3′-UTRs) of their target genes^18^. In our previous study, miR-21 was identified to be significantly overexpressed in human cervical cell lines and squamous cancer tissues and to regulate the proliferation, apoptosis and migration of HPV16-positive cervical squamous cells^19^. Research found that miR‐21 was a target of GAS5 in bladder cancer cells^20^. Therefore, the association of GAS5 and miR-21 in CC remains uncertain.

PDCD4, a target gene of miR-21, has been considered an efficient suppressive regulator in many cancers, including CC. Overexpression of PDCD4 can inhibit apoptosis and enhance chemosensitivity to cisplatin^21,22^. Research has shown that miR-21 suppression could affect cisplatin sensitivity by mediating the PTEN/PI3K/AKT and PDCD4/JNK signalling pathways in A549/DDP cells^23^. In nasopharyngeal carcinoma, PDCD4 enhances cisplatin sensitivity by stimulating the PI3K/AKT/c-jun feedback loop^24^. However, whether PDCD4 is involved in the influence of GAS5 on cisplatin sensitivity in CC has not been determined.

Our study revealed that GAS5 can bind and sequester miR‐21 and further affect the expression of PDCD4, which enhanced cisplatin sensitivity in CC. The transcription factor phosphorylated signal transducer and activator of transcription (P-STAT3) bound to the promoter region of GAS5 to transcriptionally modulate its expression. Thus, the STAT3-regulated GAS5/miR-21-5p/PDCD4 axis may have therapeutic potential for restoring the effectiveness of cisplatin-based chemotherapy in CC.

## Materials and methods

### Cell culture and tissue samples

HeLa and SiHa CC cells and 293T cells (both obtained from the American Type Culture Collection (ATCC)) were grown in Dulbecco's modified Eagle's medium (Gibco; Thermo Fisher Scientific, Inc.) supplemented with 10% foetal calf serum (Gibco; Thermo Fisher Scientific, Inc.), 100 IU/ml penicillin G and 100 mg/ml streptomycin sulfate (Sigma‑Aldrich, St. Louis, MO, USA). Cells were incubated at 37 °C in a humidified 5% CO_2_ atmosphere. The specimens used for quantitative PCR (qPCR) and for preparation of in situ hybridization (ISH) slides were harvested with informed consent from patients with CC who underwent surgery at Sun Yat-sen Memorial Hospital. The study was approved by the Institutional Review Board of Sun Yat-sen Memorial Hospital. Two independent cohorts were enrolled for this study. Cohort 1 comprised 30 paired CC tissues and adjacent non-tumour tissues obtained from patients during surgery. Cohort 2 comprised 88 paraffin-embedded CC specimens and 19 adjacent normal specimens collected from the Department of Pathology in Sun Yat-sen Memorial Hospital.

### Bioinformatic analysis

TCGA (The Cancer Genome Atlas) expression data for the lncRNA GAS5 in 306 cancer and 13 normal tissue samples were acquired from GEPIA. The expression levels of miR-21, STAT3 and PDCD4 were downloaded from UCSC Xena and SangerBox. All data are available online, and access does not require patient consent or other permissions. The use of the data does not violate the rights of any person or institution.

Potential transcription factors were predicted using the UCSC tool. After screening the target genes, the binding sites in the upstream promoter binding region in GAS5 were predicted with the JASPAR CORE database.

The potential interaction sites in GAS5 and miR-21 were predicted with the web‐based online server ViennaRNA Web Services. The interaction between miR-21 and the 3′‐UTR of PDCD4 was predicted with the online database miRDB.

### **Chromatin immunoprecipitation** (ChIP) assay

A SimpleChIP Enzymatic Chromatin IP Kit (CST) was used for the ChIP assay according to the manufacturer’s protocol. Briefly, HeLa cells were incubated with 1% formaldehyde for 10 min to generate DNA–protein cross-links. Then, cell lysates were sonicated into 100–500 bp fragments and immunoprecipitated overnight separately with anti-P-STAT3 (#9145T, Cell Signalling Technology), anti-histone H3 (the positive control) and anti-rabbit immunoglobulin G (the negative control (NC)) antibodies. After incubation with beads for 2 h, the immunoprecipitated DNA was eluted by incubation in 100 μl of ChIP elution buffer containing 10 μl of proteinase K at 65 °C for 30 min with rotation. The eluted DNA was purified and then dissolved in 50 μl of DNA elution buffer. The JASPAR CORE database was used to identify the top four binding sites between STAT3 and the genomic region 2000 bp upstream of the GAS5 sequence. Immunoprecipitated chromatin was analysed by qPCR using primers targeting the predicted binding sites. The primer sequences used for ChIP-qPCR are listed in Supplementary Table 2.

### Construction of plasmid vectors and cell lines stably transfected with GAS5

The cDNA sequence encoding GAS5 was PCR amplified and then subcloned into the vector pLVX‐IRES‐Puro. The empty pLVX‐IRES‐Puro vector was used as the control. Stably transfected cell lines were constructed as described below. HeLa and SiHa CC cells were seeded in a six-well plate. When the cells were 70–80% confluent, the GAS5 lentiviral vector (constructed by GeneChem, Shanghai, China) and HitransP G were added for infection. Forty-eight hours after lentiviral infection, cells were transferred into 10-cm culture dishes. Puromycin (4 µg/ml) was added to screen stably transfected cells. Cells were harvested 3 days after screening, and quantitative real-time polymerase chain reaction (qRT-PCR) was conducted to evaluate the transfection efficiency.

### Transfection

The miR-21‑5p mimic, si-PDCD4 and the corresponding NC sequences were purchased from GenePharma (Guangzhou, China). Briefly, CC cells were seeded in a six-well plate. When the cells were 70–80% confluent, the miR-21 inhibitor, miR-21 inhibitor NC, miR-21 mimic, miR-21 mimic NC, or si-PDCD4 at a final concentration of 20 nM was transfected into HeLa or SiHa cells using Lipofectamine™ 3000 (Invitrogen/Life Technologies, Carlsbad, CA, USA) according to the manufacturer’s instructions. For the rescue experiment, the miR-21 mimic or NC was transfected into cell lines stably transfected with GAS5. Functional experiments were performed after transfection for 48 h.

### RNA isolation and qRT-PCR

Total RNA was isolated from cell lines using TRIzol reagent (Takara Bio, Inc., Shiga, Japan), and a total of 1 μg RNA was then converted into cDNA using Prime Script RT Master Mix (Takara Bio, Inc.) according to the manufacturer’s instructions. The specific miR‑21‑5p reverse transcription primer and the U6 gene reverse transcription primers were added to the miR-21 and U6 RT-PCR samples, respectively. The expression levels of GAS5, PDCD4 and miR-21 were determined by qRT-PCR using a SYBR GREEN MIX kit (Takara Bio, Inc., Shiga, Japan) according to the manufacturer’s protocols. β-Actin was used to normalize the relative expression levels of GAS5 and PDCD4. U6 was used to normalize the relative expression level of miR-21. Fold changes in the relative expression levels of the target genes were calculated using the 2^−ΔΔCT^ method. All primers used are listed in Supplementary Table 1.

### RNA immunoprecipitation (RIP) assay

To prove that GAS5 regulated miR-21 as a competitive endogenous RNA (ceRNA), we used the Ago2-LincK RIP assay to detect the association of GAS5 with the RISC complex. Briefly, beads conjugated to an anti-AGO2 antibody (#ab557113, Abcam, Cambridge, MA, USA) or NC mouse IgG (Millipore, Bedford, MA, USA) were incubated at 4 °C overnight. Then, more than 10^7^ HeLa cells were harvested in PL buffer (100 mM KCl, 5 mM MgCl_2_, 10 mM HEPES (pH 7.0), 400 µM VRC, 1 mM DTT and 0.5% NP-40) containing 100 U/ml RNase and protease inhibitors, and 100 μl of cell lysate was incubated with magnetic beads and the antibodies described above in NT2 buffer (50 mM Tris-HCl (pH 7.4), 150 mM NaCl, 1 mM MgCl_2_, 0.05% NP-40). Subsequently, the beads were washed five times with cold NT2 buffer and incubated with proteinase K to digest proteins. RNA associated with the beads was extracted by TRIzol reagent (Takara Bio, Inc., Shiga, Japan) and detected by qRT-PCR. The miR-21 and GAS5 levels were detected by qRT-PCR.

### RNA pull-down assay

RNA pull-down assays were performed to prove the interaction between GAS5 and miR-21. Full‐length GAS5 was transcribed in vitro with T7 RNA Pol II (Promega, Madison, WI) and labelled with biotin using Biotin RNA Labeling Mix (Roche, USA). An oligo probe (Bio-NC) was used as the NC. HeLa cells were fixed with formaldehyde to cross-link RNA and then suspended in 1 ml of lysis buffer (20 mM Tris-HCl, 200 mM NaCl, 2.5 mM MgCl_2_·6H_2_O, 0.05% IGEPAL) containing RNase and protease inhibitors. Three micrograms of biotinylated RNA (Bio-GAS5, Bio-NC) was incubated upside down with Streptavidin Magnetic Beads (#88816, Invitrogen) at room temperature for 2 h. Then, we added 500 μl of cell lysate supernatant to the magnetic beads for another 2 h of incubation at 4 °C. After sequential elution with solution A (100 mM NaOH, 50 mM NaCl) and solution B (100 mM NaCl) three times, the magnetic beads were resuspended in 200 μl of lysis buffer and rotated at room temperature for 2 h to release the formaldehyde cross-linked RNA in the sample. The RNA–RNA complexes were purified using TRIzol reagent (Takara). The miR-21 level in the RNA–RNA complexes was detected by qRT-PCR.

### Dual-luciferase reporter assay

The wild-type (WT) PDCD4 3′‐UTR fragment was cloned downstream of the Renilla luciferase gene in the psiCHECK-2 vector (Promega, Madison, WI). Mutants were generated by mutating base pairs in the miR-21-binding sequences. WT miR‑21 with GAS5-binding sequences and mutant‑type (MUT) miR‑21 with no GAS5-binding sequences were cloned using the same procedure. To study the relationship between GAS5 and miR‑21, we used Lipofectamine 3000 (Thermo Fisher Scientific, Inc.) to co-transfect the WT or MUT miR-21 luciferase reporter with a GAS5 overexpression plasmid or its control vector into 293T cells. To clarify the targeting relationship between miR-21 and PDCD4, we co-transfected the WT or MUT-PDCD4 3′‑UTR luciferase reporter plasmid with the miR-21 mimic or the corresponding NC into 293T cells. Cells were harvested 48 h after transfection, and Renilla and firefly luciferase activities were analysed using the Dual-Luciferase® Reporter Assay System (#E2920, Promega). Renilla and Firefly luciferase activity values were normalized to the firefly luciferase activity value for each transfected well. All assays were performed in triplicate.

### Detection of apoptosis

After transfection for 48 h, SiHa cells were treated with 15 µg/ml cisplatin for 48 h. The cells were then digested with EDTA-free trypsin and washed twice with cold phosphate-buffered saline (PBS). After incubation with Annexin-V and PI for 10 min at room temperature, flow cytometry was performed using a flow cytometer (FACSCalibur, Becton Dickinson, Franklin Lakes, NJ, USA).

### Western blot analysis

After treatment, cells were harvested and lysed in ice-cold RIPA lysis buffer (FD, Hangzhou, China) containing a protease inhibitor cocktail and phosphatase inhibitor cocktail on ice for 30 min. After centrifugation at 13,000 × *g* for 30 min at 4 °C, the protein content in the supernatant was quantified using a BCA protein assay kit (CWBIO, Beijing, China). The protein samples were then heated at 100 °C for 10 min, and equal amounts (30 µg) of protein were separated by 8 and 12% sodium dodecyl sulfate denaturing polyacrylamide gel electrophoresis and transferred onto 0.22-µm polyvinylidene difluoride membranes (Millipore, Bedford, MA, USA). The membranes were blocked with 5% non-fat milk for 1 h at room temperature and then incubated first with rabbit anti-human antibodies specific for PDCD4 (1:2000 dilution; Cell Signalling Technology), PARP (1:1000 dilution; Cell Signalling Technology), caspase-3 (1:1000 dilution; Cell Signalling Technology), STAT3 (1:1000 dilution; Cell Signalling Technology) and P-STAT3 (1:1000 dilution; Cell Signalling Technology) at the recommended dilutions and with mouse anti-human antibodies specific for GAPDH (1:5000 dilution; FD) overnight at 4 °C and then with horseradish peroxidase (HRP)-conjugated goat anti-rabbit IgG (1:10,000) or goat anti-mouse IgG (1:10,000) for 2 h at 37 °C. After the membranes were washed in PBS containing Tween 20, signals were visualized with an ECL kit (Millipore, USA) and analysed using a FluorChem FC2 Imaging System (Alpha Innotech, San Leandro, CA, USA). The protein expression levels were normalized to those of GAPDH as the internal control.

### In situ hybridization

The expression level of GAS5 in tissues was measured with digoxigenin-labelled antisense oligonucleotide probes. Slides of CC tissue were dewaxed and rehydrated. After treatment with 3% H_2_O_2_ at room temperature for 10 min, tissue slices were digested with pepsin diluted with 3% citric acid to expose RNA. After incubation with prehybridization buffer for 2 h at 37 °C, the slices were hybridized with the GAS5 probe (Genebio, Guangzhou, China; 1 µM) at 37 °C overnight. Sections were then washed with a gradient-diluted SSC solution at 37 °C for 15 min and subsequently incubated with biotinylated mouse anti-digoxigenin antibodies for 60 min at 37 °C. After incubation with both SABC and biotinylated peroxidase at 37 °C for 20 min, hybridization signals were visualized with 3,3′-diaminobenzidine (DAB) chromogenic substrate (Dako). The reaction was stopped by washing with distilled water for 5 min. Slides were counterstained with haematoxylin, dehydrated with ethyl alcohol, sealed with neutral resins and imaged. The GAS5 probe sequence was as follows: 5′-CCATAAGGTGCTATCCAGAGC-3′. Two pathologists evaluated the ISH scores in a blinded manner. The staining intensity of GAS5 was scored on a scale of 0–3 as follows: 0 (negative), 1 (weak), 2 (moderate) and 3 (strong). For statistical analysis, we divided the samples into two groups: slides with scores of 2 and 3 were classified into the high GAS5 expression group and those with scores of 0 and 1 were classified into the low-expression group.

### Xenograft assay in nude mice

Six female BALB/c nude mice (5 weeks old) were obtained from the Guangdong Medical Laboratory Animal Center and raised in the Department of Laboratory Animal Science, Sun Yat-sen University (Guangzhou, China). All animal experiments were conducted in accordance with the Declaration of Helsinki and were approved by the Research Animal Resource Center of Sun Yat-Sen University (application number 2019000253). Mice were randomly assigned to two groups, with three mice in each group. To establish a subcutaneous model, SiHa cells with GAS5 overexpression and corresponding control cells (stably transduced with lentiviral vectors) were resuspended in PBS at a concentration of 10^7^ cells/100 μl. Mice were then subcutaneously injected in the right flank with 100 μl of cell suspension to induce tumour formation. When the tumour sizes reached 50 mm^3^, the mice were injected weekly with 2 mg/kg cisplatin. Two weeks after the first injection, the mice were killed, and the tumours were excised. Tumour tissues were harvested for tissue processing. A 4‑mm portion of each tumour was fixed with paraffin, and immunohistochemistry (IHC) was performed to detect proteins. Tumour sizes were measured weekly, and the volumes (in cubic millimetres) were calculated according to the following equation: width^2^ × length × 0.5. Length and width refer to the longest and shortest diameters, respectively.

### Immunohistochemistry

The protein expression levels of PDCD4 in subcutaneous xenograft mouse tumours were assessed by IHC with the corresponding anti-PDCD4 antibody (1:200 dilution; #9535, Cell Signalling Technology). The protein expression levels of P-STAT3 in CC tissues were assessed by IHC with the corresponding anti-P-STAT3 antibody (1:100 dilution; #4113S, Cell Signalling Technology). The tumours were fixed, embedded in paraffin and sectioned into 4-μm-thick sections. After deparaffinization and rehydration, the sections were incubated with 3% H_2_O_2_ for 10 min and then with 10% normal goat serum for 15 min at room temperature to block endogenous peroxidase activity and non-specific antigen binding. Histological sections were incubated with primary antibodies at 4 °C overnight and then rinsed three times with PBS. The sections were incubated with an HRP-conjugated secondary antibody for 30 min at room temperature. To visualize staining, slides were incubated in Tris-HCl buffer containing DAB and 0.1% H_2_O_2_ and then counterstained with haematoxylin.

### Subcellular fractionation

Nuclear and cytosolic fractions were separated using a PARIS™ Kit (#AM1921, Thermo Scientific) based on the supplier’s recommendation. One billion HeLa and SiHa cells were collected for one test. RNA was extracted, and qRT-PCR was performed to assess the GAS5 levels in the nuclear and cytoplasmic fractions with normalization to MALAT1 (nuclear control) and β-actin (cytoplasmic control).

### Statistical analysis

Data are presented as the mean values ± standard deviations (means ± SDs) in this study. GraphPad Prism 7.0 (La Jolla, CA) and SPSS 20.0 (SPSS, USA) were used to conduct statistical analyses. Student’s *t-*test was used to analyse gene expression differences. Multiple comparisons were conducted by one-way ANOVA. Estimation of survival differences was performed using the Kaplan–Meier method and log-rank test. Univariate and multivariate Cox regression analyses were used to identify potential prognosis-associated factors. A chi-square test was performed to analyse clinicopathological characteristics. The Pearson correlation coefficient was used to evaluate correlations. *P* values less than 0.05 were considered statistically significant.

## Results

### GAS5 expression is downregulated in cervical tissues

**Fig. 1:**
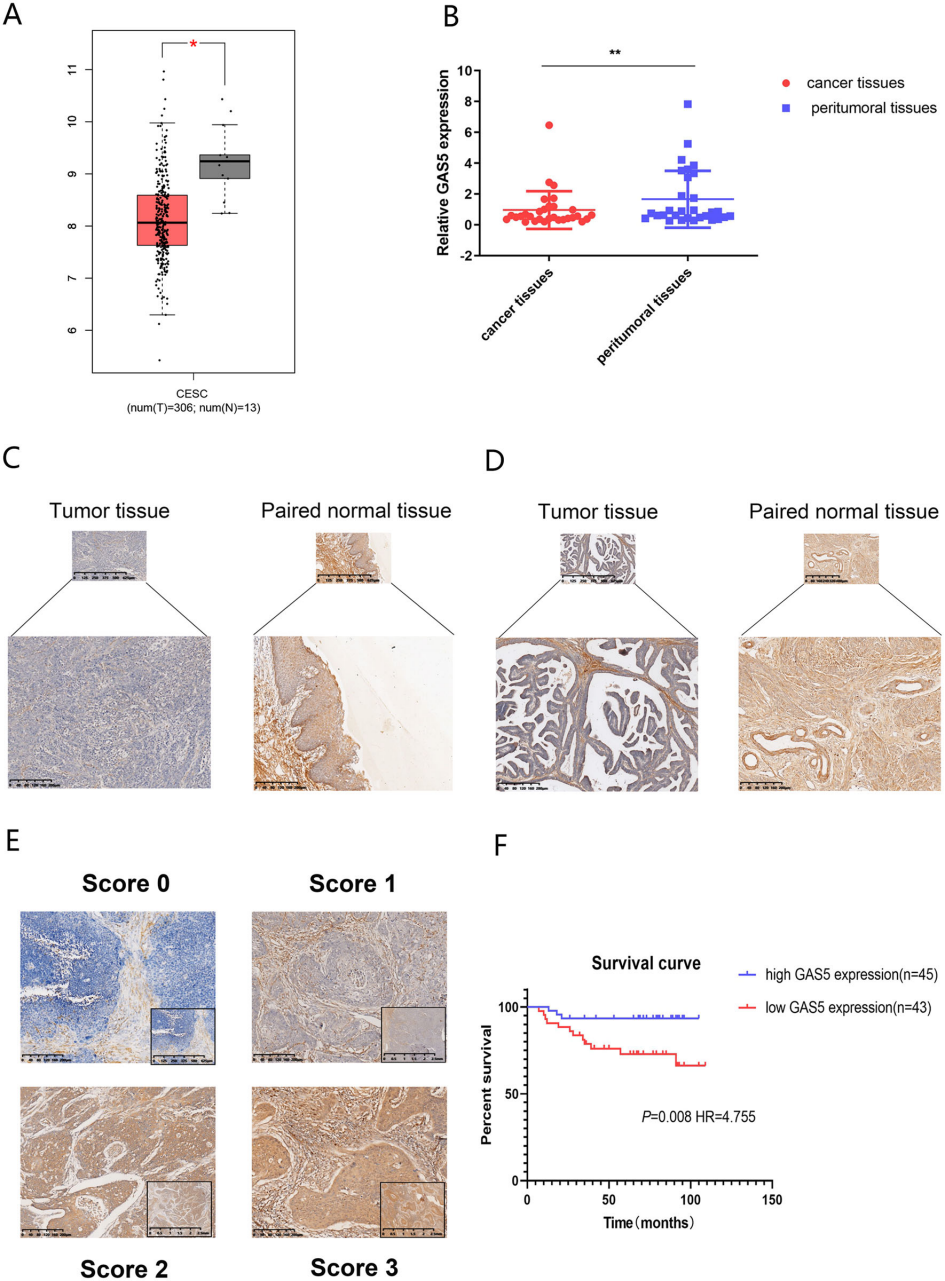
GAS5 is notably downregulated in CC tissues and cell lines. **a** The expression level of GAS5 in the TCGA database analysed via GEPIA. **b** GAS5 was downregulated in CC tissues compared to corresponding normal tissues as detected by qPCR in cohort 1. **c** Representative images of GAS5 expression in cervical squamous carcinoma tissues and adjacent normal tissues as detected by ISH. **d** Representative images of GAS5 expression in cervical adenocarcinoma tissues and adjacent normal tissues as detected by ISH. **e** Representative GAS5 staining patterns in cohort 2. **f** Low GAS5 expression was correlated with poor overall survival in cervical cancer.

To determine the enrichment of GAS5 in CC, we investigated the expression level of GAS5 in the TCGA database via GEPIA, which indicated a low level of GAS5 in cancer tissues compared to adjacent normal tissues (Fig. 1a). To confirm this result, we examined the expression level of GAS5 in 30 CC tissues and adjacent noncancerous tissues (cohort 1). The qPCR results revealed that GAS5 was expressed at low levels in tumour tissues compared to peritumoral tissues (Fig. 1b). In addition, we designed and synthesized ISH probes for GAS5 and used ISH to examine GAS5 expression in 88 CC tissues and 19 peritumoral tissues (cohort 2). GAS5 staining was observed mainly in the cytoplasm. Consistent with the qRT-PCR results, the expression of GAS5 was reduced in most tumour tissues compared to peritumoral tissues in cervical squamous carcinoma tissues (Fig. 1c) and cervical adenocarcinoma tissues (Fig. 1d). Moreover, the degree of GAS5 expression differed among CC specimens (Fig. 1e). After grouping according to the scores, we further summarized the relationships between GAS5 expression and the clinicopathological features of CC patients, as shown in Table 1. GAS5 expression was significantly correlated with vaginal recurrence or metastasis (*P* < 0.01), chemotherapy (*P* < 0.01) and age (*P* < 0.05). Importantly, Kaplan–Meier analysis illustrated that low GAS5 expression was correlated with poor OS (Fig. 1f) (*P* = 0.008, hazard ratio (HR) = 4.775). Univariate analysis indicated that GAS5 (HR = 4.755, 95% confidence interval (CI) = [1.340–16.875], *P* = 0.016) was a potent independent prognostic indicator for CC patients. However, the *P* values in multivariate analyses indicated no statistical significance (Table 2). Logistic regression analysis showed that compared to high GAS5 expression, low GAS5 expression increased the risk of recurrence or metastasis, which accounted for the above result (Supplementary Table 3). In summary, we confirmed that GAS5 was connected with prognosis and chemotherapeutic efficacy in CC.

**Table 1:** Association of GAS5 expression with clinicopathological features from CC patients.

Features	*n*	High	Low	*P*
**Age(years)**				
≥50	32	21	11	0.048*
<50	56	24	32	
**FIGO stage**				
I and II	19	7	12	0.199
III and IV	69	38	31	
**Tumour sizes**				
≥3	38	18	20	0.667
<3F	50	27	23	
**LN status**				
Positive	20	7	13	0.129
Negative	68	38	30	
**Histological grade**				
Low differentiation	30	15	15	0.878
Moderate and high differentiation	58	30	28	
**Vaginal recurrence or metastasis**				
Present	35	12	23	0.016^*^
Absent	53	33	20	
**Chemotherapy**				
Yes	43	12	31	0.001^*^
No	45	33	12	

*P < 0.05.

**Table 2:** Univariate and multivariate Cox regression analysis for OS in CC patient.

Variable	Univariate analysis		
	HR	95% CI	*P* value
**Univariate analyses**			
Age(<50 vs ≥50)	1.989	0.633–6.252	0.239
FIGO stage (I and II vs III and IV)	2.303	0.819–6.471	0.114
Tumour sizes ≥3 vs <3	2.835	0.968–8.304	0.057
LN status (positive vs negative)	0.54	0.184–1.581	0.261
Histological grade (low differentiation vs moderate and high differentiation)	0.36	0.143–0.908	0.031*
Vaginal recurrence or metastasis (present vs absent)	33.266	4.313–256.574	0.001*
GAS5 expression (high vs low)	4.755	1.340–16.875	0.016*
**Multivariate analyses**			
Vaginal recurrence or metastasis (present vs absent)	34.481	4.438–267.891	0.001
Histological grade (low differentiation vs moderate and high differentiation)	0.287	0.101–0.816	0.019
GAS5 expression (high vs low)			0.175

*HR* hazard ratio, *CI* confidence interval.

*P < 0.05.

### GAS5 is transcriptionally regulated by P-STAT3 in CC

**Fig. 2:**
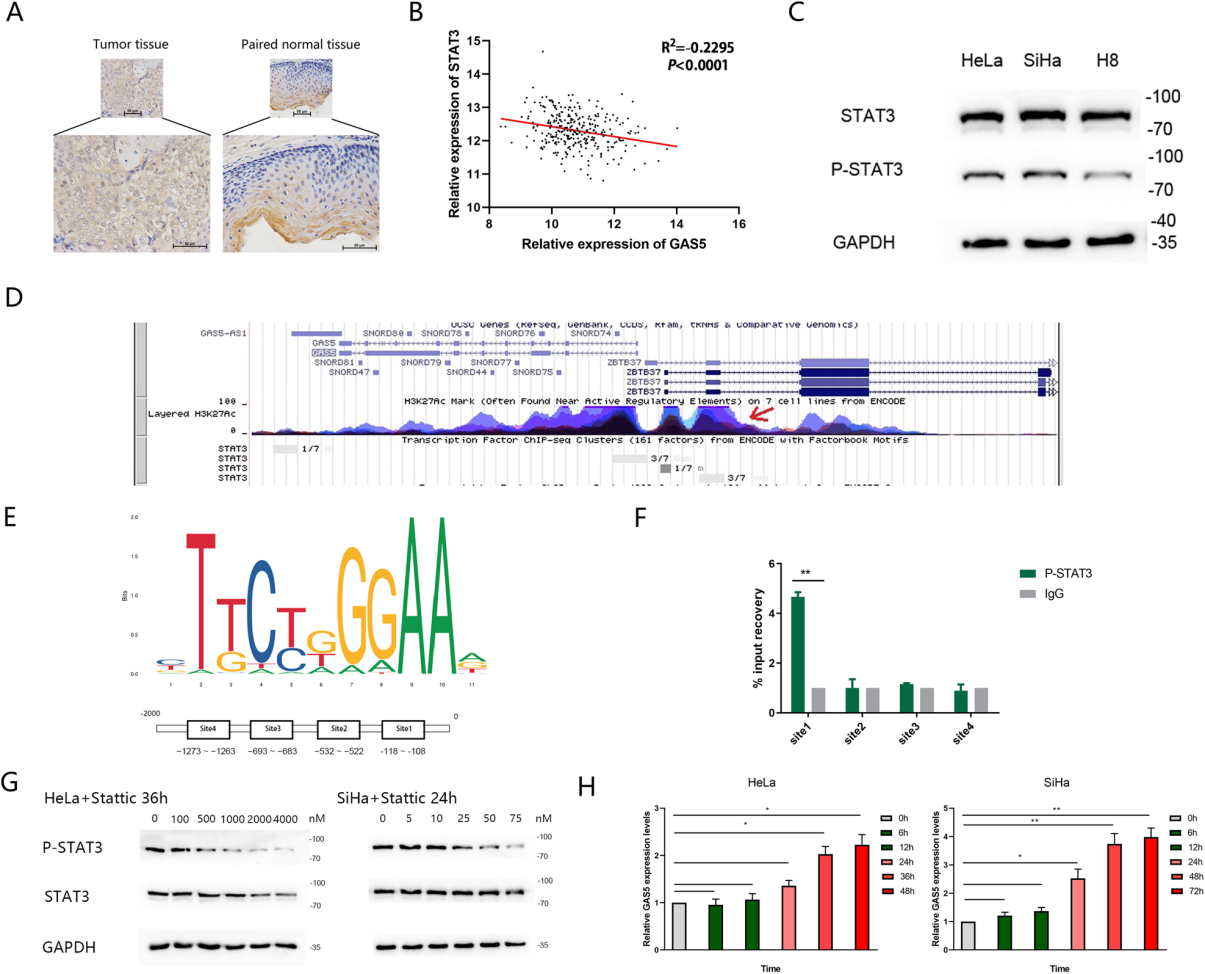
P-STAT3 binds with the promoter of GAS5 and modulates its transcriptional level. **a** Immunohistochemical staining for P-STAT3 in cervical cancer tissues and adjacent normal tissues (magnification, ×200). Scale bar, 50 μm. **b** The correlation between STAT3 and GAS5 expression in CC tissues from the TCGA database was analysed by Pearson correlation analysis. **c** Western blot analysis showed that the expression levels of P-STAT3 in CC cells were higher than those in normal cervical cell, whereas the total STAT3 level did not differ. **d** UCSC database analysis revealed that the promoter of GAS5 had high enrichment of STAT3. **e** The binding motif of the transcription factor STAT3 was predicted via the JASPAR CORE database. **f** A ChIP assay demonstrated P-STAT3 binding to site 1 (−118 to −108) in the GAS5 promoter region. **P < 0.01 (Student’s *t-*test). **g** Western blot analysis showed that the P-STAT3 level changed dose-dependently under treatment with Stattic, whereas the total STAT3 level remained unchanged at Stattic concentrations of 0–50 nM for SiHa cells and 0–1000 nM for HeLa cells. GAPDH was used as the control. **h** A decline in the P-STAT3 level was accompanied by an increase in the GAS5 level. Total RNA was isolated from SiHa cells treated with 50 nM Stattic and HeLa cells treated with 1000 nM Stattic for different durations. GAS5 levels were examined by qRT-PCR. β-Actin was used as the input control. The fold change was calculated with respect to the control by the 2^−ΔΔCt^ method. The data are shown as the means ± SDs (*n* = 3). *P value <0.05 compared to untreated cells.

Inhibition of STAT3 is reported to sensitize tumour cells to cisplatin-induced apoptosis^25^. Immunohistochemical found that P-STAT3 expression was higher in CC tissues than that in adjacent normal tissues (Fig. 2a). TCGA database analysis showed that STAT3 expression was negatively correlated with GAS5 expression in CC tissues (Fig. 2b). In addition, the expression level of P-STAT3 was higher in CC cells than in normal cervical cells, whereas the total STAT3 level did not differ (Fig. 2c). As STAT3 enters the nucleus after phosphorylation for transcriptional regulation, we sought to determine whether the reduction of GAS5 was related to the phosphorylation of STAT3. Exploring the probable mechanisms using genome bioinformatic analysis, we found that STAT3 was a potential transcription factor for GAS5 (Fig. 2d). We obtained the binding motif of STAT3 from JASPAR and selected four binding sequences for a ChIP experiment (Fig. 2e). As shown in Fig. 2f, P-STAT3 was enriched at site 1 in the promoter region of the GAS5 gene in HeLa cells. To further evaluate the effect of P-STAT3 on GAS5 expression, we inhibited STAT3 phosphorylation with Stattic (Selleck Chemicals, America) at a dose of at least 5 nM in HeLa cells for 36 h and in SiHa cells for 24 h. Western blot analysis showed that for HeLa cells, the decline in P-STAT3 exhibited a dose-dependent tendency at concentrations of 50–1000 nM, whereas the total STAT3 level remained unchanged. However, at 2000–4000 nM concentration, a simultaneous decline in total STAT3 was observed. For SiHa cells, the dose for a decline in P-STAT3 and no change in total STAT3 was 5–50 nM. Simultaneous decline in total STAT3 was observed from 75 nM. (Fig. 2g) Thus, we selected 1000 nM Stattic for HeLa cells and 50 nM Stattic for SiHa cells to evaluate the effect of STAT3 phosphorylation on GAS5 expression. After adding Stattic, we extracted RNA at 0, 6, 12, 24, 36, 48 and 72 h. The qPCR results showed that GAS5 expression was gradually upregulated after 36 h for HeLa and 24 h for SiHa (Fig. 2h).

### GAS5 is localized mainly in the cytoplasm and acts as a ceRNA in CC by sponging miR-21

Recently, several mechanisms by which lncRNAs regulate transcription have been reported.

**Fig. 3:**
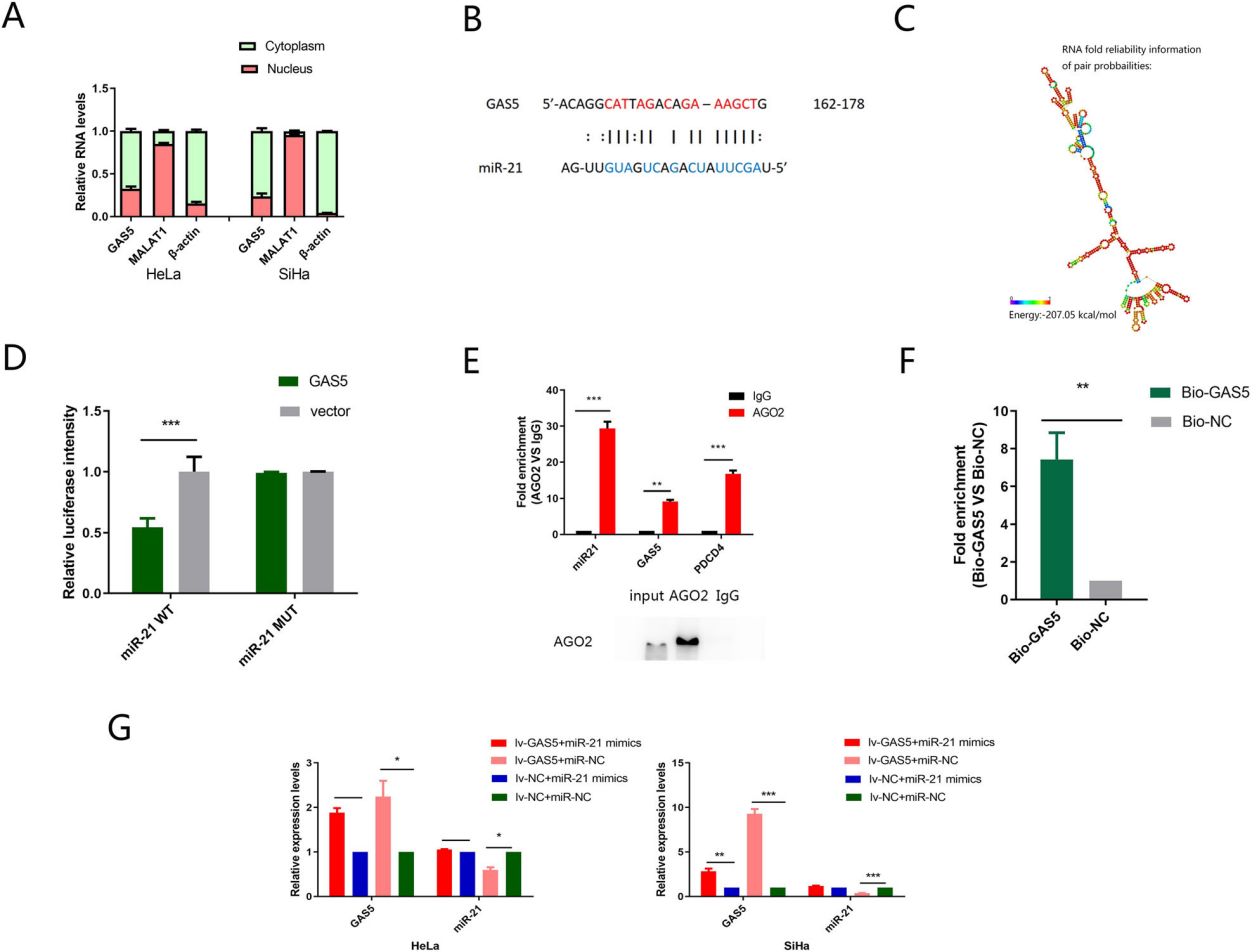
GAS5 physically associates with miR-21. **a** Fractionation of HeLa and SiHa cells followed by qRT-PCR. β-Actin served as the cytoplasmic mRNA control. MALAT1 served as the nuclear RNA control. The results showed that GAS5 was mainly located in the cytoplasm. **b** Bioinformatics manners predicted the potential binding sites within GAS5 and miR-21. **c** RNA secondary structure of GAS5 predicted by the Vienna RNAfold server. **d** Compared to co-transfection of the GAS5 plasmid and miR-21 MUT plasmid, co-transfection of the GAS5 plasmid and miR-21 WT plasmid strongly decreased luciferase activity. **e** RIP assay showed that miR-21 and GAS5 or PDCD4 expression were significantly enriched in the Ago2 pellet compared to the IgG control. **f** An RNA pull-down assay followed by qRT-PCR was performed to assess the interaction between GAS5 and miR-21 using biotin-labelled GAS5. The miR-21 level in the biotin-GAS5 group was compared to that in the biotin-NC group. U6 was used as the non-specific control. **g** Rescue experiments showed that the miR-21 mimic could rescue GAS5 overexpression, and that GAS5 also downregulated the expression of miR-21. The data are shown as the means ± SDs (*n* = 3). *P < 0.05, **P < 0.01, ***P < 0.001.

By evaluating the subcellular distribution of GAS5, we found that GAS5 was localized mainly in the cytoplasm (Fig. 3a). Considering the role of miR-21, we hypothesized that GAS5 exerted its function by competitively binding with miR-21 as a ceRNA. We predicted the potential binding sites of miR-21 with GAS5 through ViennaRNA Web Services (Fig. 3b). The secondary structure of GAS5 is shown in Fig. 3c. A dual-luciferase reporter assay was used to verify the binding sites between GAS5 and miR-21. GAS5 overexpression markedly attenuated the luciferase reporter activity of WT miR-21 (Fig. 3d).

In addition, we performed an RIP assay using an anti-AGO2 antibody to further confirm this effect. GAS5, miR-21 and PDCD4 could bind to AGO2, a key component of the RISC complex (Fig. 3e), indicating that GAS5 or PDCD4 formed an RISC complex with miR-21. Subsequently, we used in vitro transcribed biotin-labelled GAS5 to pull-down endogenous miRNAs in HeLa cells and found that miR-21 was significantly enriched compared to biotin-labelled RNA oligo (Fig. 3f). These results demonstrated that GAS5 was physically associated with miR-21 and might function as a ceRNA to affect the target gene of miR-21.

Moreover, rescue experiments were performed to further verify these effects. We used miR-21 mimics to upregulate miR-21 in HeLa and SiHa cell lines stably transfected with GAS5. The expression levels of miR-21 and GAS5 were measured by qRT-PCR. GAS5 overexpression was abrogated by miR-21 mimics, and GAS5 also downregulated the expression of miR-21 (Fig. 3g).

### PDCD4 is a direct target gene of miR-21

**Fig. 4:**
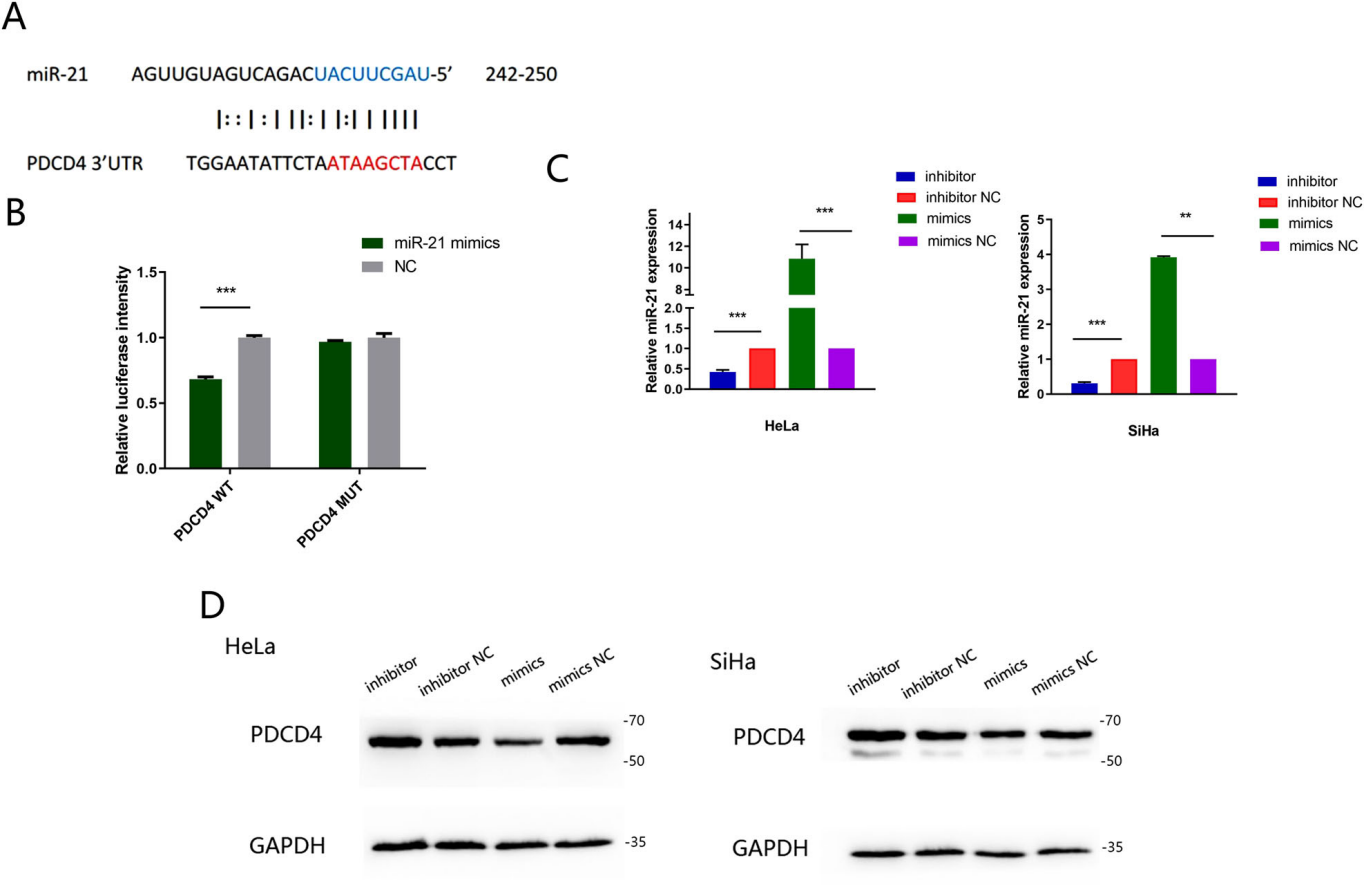
PDCD4 is a direct target gene of miR-21. **a** The binding site of PDCD4 in miR-21 was predicted via miRDB. **b** The indicated WT- or MUT-PDCD4 3′-UTR luciferase vectors and miR-21 mimic/NC were co-transfected into 293T cells. Transfection with the miR-21 mimic significantly reduced the luciferase activity of the PDCD4-WT reporter. **c** MiR-21 expression was knocked down by transfection with the miR-21 inhibitor and overexpressed by transfection with the miR-21 mimic. **d** Relative protein levels of PDCD4 in HeLa and SiHa cells transfected with the miR-21 inhibitor, miR-21 inhibitor NC, miR-21 mimic or miR-21 mimic NC. The data are shown as the means ± SDs (*n* = 3). **P < 0.01, ***P < 0.001.

Through the RIP assay, we discovered that PDCD4 existed in the RISC complex. Then, we revealed a site for miR-21 binding with the 3′-UTR of PDCD4 by miRDB analysis (Fig. 4a). To further verify this site, we inserted fragments of the PDCD4 3′-UTR containing WT or MT miR-21-binding sites into the psiCHECK-2 vector and co-transfected them with the miR-21 mimic. The miR-21 mimic obviously attenuated the relative luciferase activity of the WT PDCD4 reporter, while the MT PDCD4 reporter was unaffected (Fig. 4b). To confirm whether PDCD4 is a target gene of miR-21, we measured PDCD4 expression in HeLa and SiHa cells after transfection with the miR-21 mimic or inhibitor (Fig. 4c). PDCD4 expression was significantly downregulated in miR-21 mimic-treated cells compared to NC-treated cells and markedly upregulated after transfection with the miR-21 inhibitor compared with the NC inhibitor (Fig. 4d).

### MiR-21-5p reverses the influence of GAS5 on apoptosis in CC through the PDCD4 pathway

**Fig. 5:**
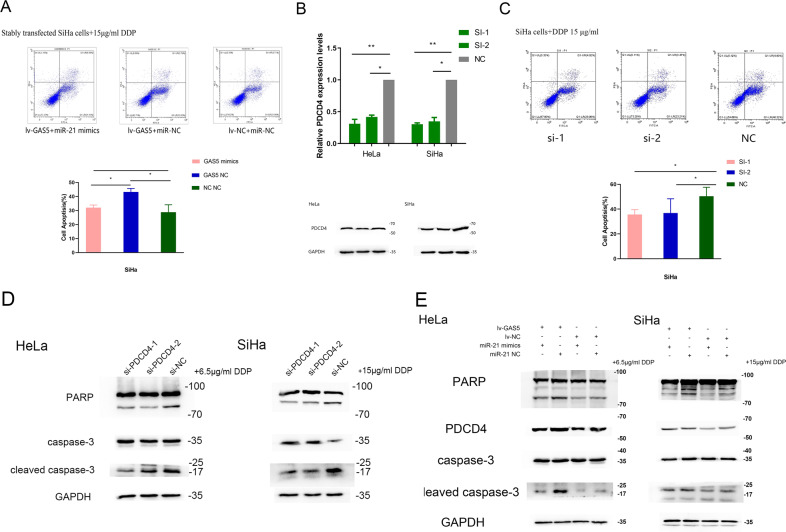
The miR-21 mimic reverses the apoptosis-promoting function of GAS5 through the PDCD4 pathway. **a** Flow cytometric analysis showed that introduction of the miR-21 mimic attenuated the apoptosis-promoting effect of GAS5 in SiHa cells. The lower right quadrant of each apoptosis diagram is the early apoptotic cells and the upper right quadrant is the late apoptotic cells. The percentages of apoptotic cells were the sum of the early and late apoptotic cells and were compared among the groups. **b** Si-PDCD4 was transfected into HeLa and SiHa cells to achieve PDCD4 knockdown. The transfection efficiency was verified using RT-PCR and western blotting. **c** Flow cytometric analysis showed that si-PDCD4 inhibited the apoptosis of SiHa cells. **d** Western blot analysis showed that knockdown of PDCD4 reduced the levels of cleaved PARPγ and cleaved caspase‐3 after treatment with cisplatin for 48 h in HeLa and SiHa cells. **e** Western blot analysis revealed that introduction of the miR-21 mimic attenuated the effect of GAS5 on PDCD4, cleaved PARPγ and cleaved caspase‐3 levels in CC cells. The data are from triplicate experiments and are presented as the means ± SDs. *P < 0.05 and **P < 0.01.

We confirmed that GAS5 promoted apoptosis in CC in our previous research. Flow cytometric analysis revealed that overexpression of GAS5 dramatically increased the apoptosis rate in GAS5 stably transfected SiHa cells, while transfection with the miR-21 mimic markedly reversed this effect (Fig. 5a). We therefore hypothesized that GAS5 regulated apoptosis via miR-21-5p in CC cells.

PDCD4, a target gene of miR-21, is related to apoptosis. We silenced PDCD4 and then determined the apoptosis rate through flow cytometric analysis. Moreover, the apoptosis-related proteins cleaved PARP and cleaved caspase-3 were also evaluated. The transfection efficiency results demonstrated that si-PDCD4 induced a significant decrease in PDCD4 expression (Fig. 5b). The flow cytometry results indicated that silencing PDCD4 reduced the apoptosis rate in SiHa cells (Fig. 5c). In order to detect the apoptosis by western blot, we treated cells with 15 µg/ml cisplatin (SiHa cells) or 6.5 µg/ml cisplatin (HeLa cells) for 48 h after transfection for 48 h. Analysis of protein expression showed that the levels of cleaved caspase-3 and cleaved PARP were reduced after PDCD4 silencing (Fig. 5d).

Based on the above results, we further investigated whether GAS5 regulated cell apoptosis through the PDCD4 pathway and whether cell apoptosis induced by GAS5 expression could be rescued by the miR-21 mimic. Western blotting showed that the increase of PDCD4 in CC cells stably overexpressing GAS5 was restored by the miR-21 mimic, and after cisplatin treatment, the increases of cleaved caspase-3 and cleaved PARP presented the same trends (Fig. 5e).

These results indicated that GAS5 probably promoted apoptosis in CC by competitively binding to miR-21 and indirectly regulating the PDCD4 pathway.

### Expression of PDCD4 in CC tissues and the connection among GAS5, miR-21 and PDCD4

**Fig. 6:**
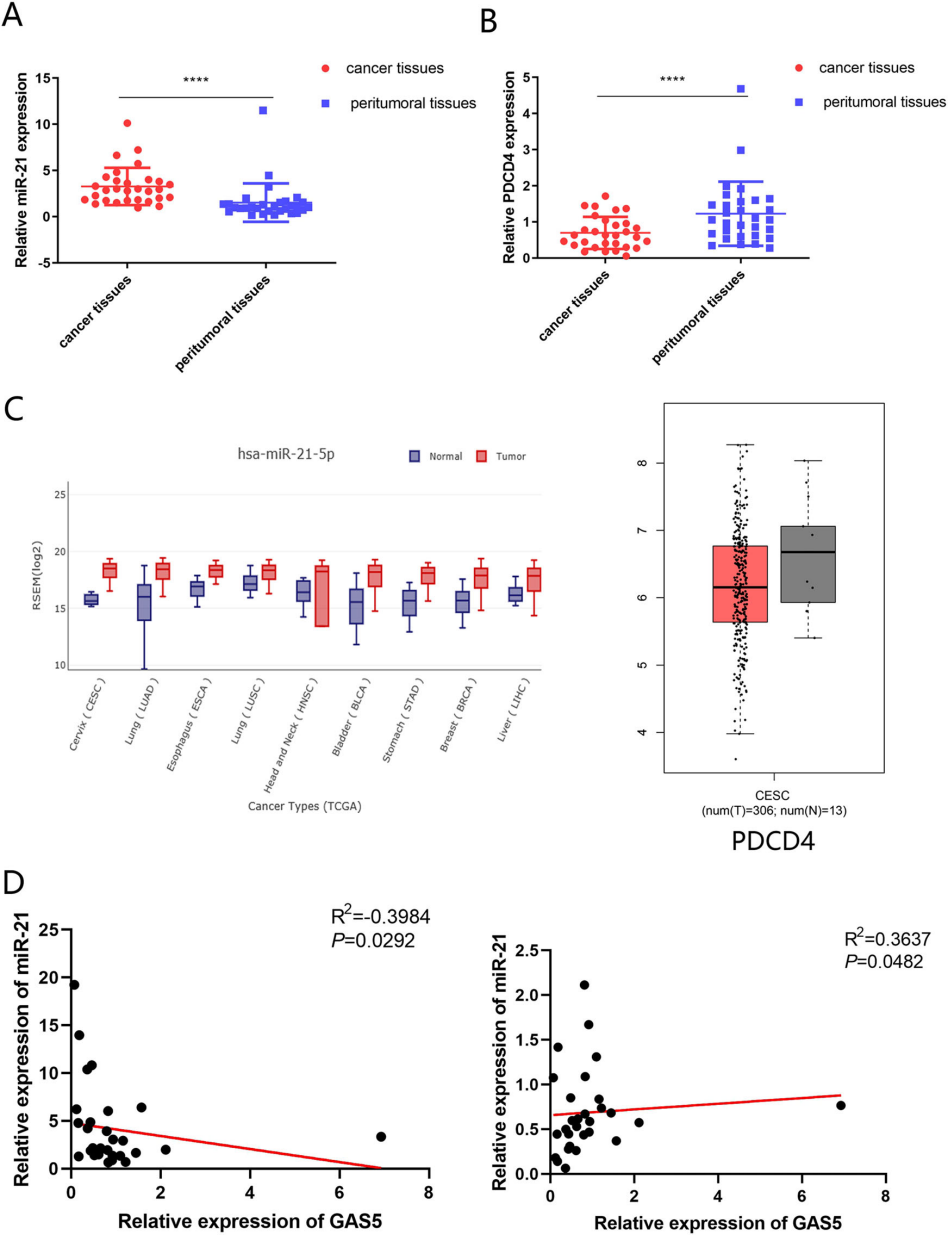
PDCD4 expression in CC tissues and the connection between GAS5, miR-21 and PDCD4. **a** MiR-21 was highly expressed in tumour tissues compared to peritumoral tissues as determined by qPCR in cohort 1. **b** PDCD4 was expressed at low levels in tumour tissues compared to peritumoral tissues as detected by qPCR in cohort 1. **c** PDCD4 expression levels in CC tissues and paired non-tumour tissues from the TCGA database and miR-21 expression levels in CC tissues and paired non-tumour tissues in GEDS. **d** The Pearson correlations between GAS5 and miR-21 or GAS5 and PDCD4 were measured in 30 pairs of CC tissues.

To validate the above results, we examined the expression levels of miR-21 and PDCD4 in cohort 1 by qPCR. In contrast to GAS5, miR-21 was highly expressed in tumour tissues compared to peritumoral tissues (Fig. 6a), while the trend in PDCD4 expression was the same as that in GAS5 expression (Fig. 6b). TCGA database analysis further confirmed our results. We investigated the PDCD4 expression level in the GEPIA database and the miR-21-5p expression level in GEDS, which showed a low level of PDCD4 and a high level of miR-21-5p in cancer tissues compared with normal tissues (Fig. 6c). Pearson rank correlation analysis of the relationship between GAS5 expression and miR-21 expression in CC patients in the TCGA database indicated a significant negative correlation, while the relationship between GAS5 expression and PDCD4 expression indicated a positive correlation (Supplementary Table 4). In addition, a negative correlation was observed for the relationship between GAS5 expression and miR-21 expression while a positive correlation was observed between GAS5 expression and PDCD4 expression (*P* < 0.05) in CC patients of cohort 1 (Fig. 6d). In summary, GAS5 expression was negatively correlated with miR-21 expression and positively associated with PDCD4 expression.

### GAS5 enhances the cisplatin sensitivity of CC in vivo

**Fig. 7:**
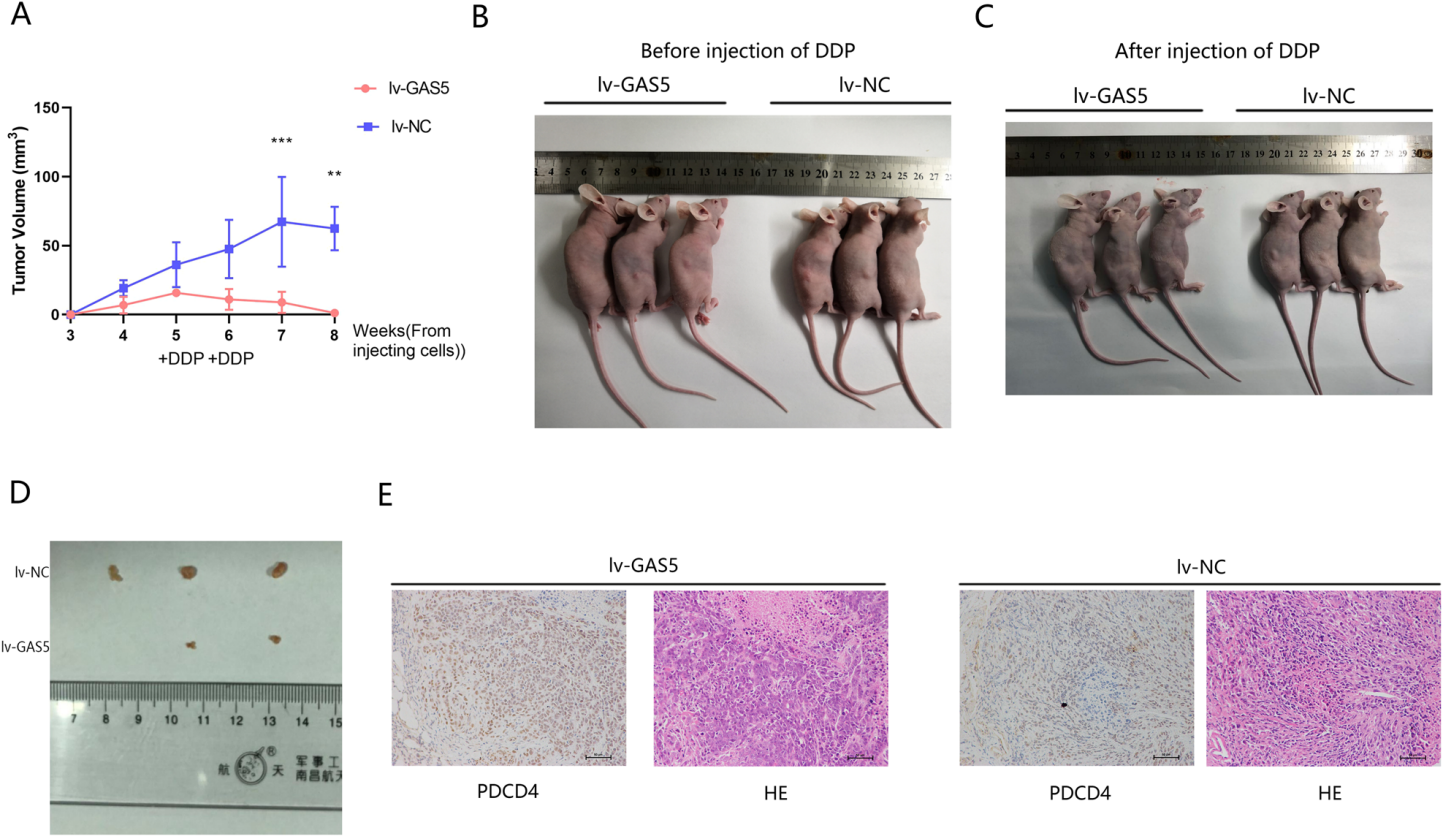
GAS5 increases the cisplatin sensitivity of CC cells in vivo. **a** Tumour sizes were measured weekly, and volumes were calculated according to the following equation: width^2^ × length × 0.5. *P < 0.05, Student’s *t*-test. The data are shown as the means ± SDs (*n* = 3). *P < 0.05, **P < 0.01, ***P < 0.001. **b** Mice were anaesthetized and photographed two weeks after tumorigenesis. **c** Mice were sacrificed and photographed at the eighth week after cell injection. **d** Tumour sizes in the lentivirus-GAS5 group were significantly lower than those in the lentivirus-NC group. One tumour in the lv-GAS5 group could not be found. **e** Xenograft tumour sections were subjected to haematoxylin and eosin (H&E) staining as well as immunohistochemical staining for PDCD4 (magnification, ×200). Scale bar, 50 μm.

To further investigate the function of GAS5 in mediating the cisplatin sensitivity of CC in vivo, GAS5 lentivirus-transfected cells were injected into BALB/c nude mice to establish a xenograft model. One million SiHa cells were injected subcutaneously into the backs of nude mice, and tumours formed in most mice 4 weeks after cell injection. At the fifth week after injecting cells, we anaesthetized the mice and photographed them in groups (Fig. 7b). Then, we injected cisplatin intraperitoneally once a week for 2 weeks to mimic chemotherapy in vivo. Tumour volumes were measured weekly until sacrifice. As expected, the tumour volumes in the lentivirus-GAS5 group were significantly smaller than those in the lentivirus-NC group. After injecting cisplatin, the tumour volume in the lentivirus-GAS5 group even showed a declining trend, while the tumour volume in the lentivirus-NC group increased (Fig. 7a). In sharp contrast, the tumours in the lentivirus-GAS5 group were invisible at the eighth week in vivo (Fig. 7c). Then, we sacrificed the mice and removed the tumours. A significant decrease in the tumour volume was observed macroscopically in the lentivirus-GAS5 group, and one of the tumours had even disappeared (Fig. 7d). Additionally, high PDCD4 expression was observed in the GAS5 overexpression group compared with the NC group as detected by IHC (Fig. 7e).

In summary, GAS5 significantly enhanced the cisplatin sensitivity of CC in vivo by upregulating PDCD4 expression.

## Discussion

Cisplatin is one of the most widely used antineoplastic drugs for the treatment of numerous human cancers, including bladder, head and neck, lung, ovarian and testicular cancers^26,27^. A major reason for the failure of cisplatin treatment is drug resistance^28^. Resistance to cisplatin, an important limiting factor affecting treatment efficacy, survival and prognosis, can easily lead to distant metastasis and relapse in CC^29^.

Numerous recent studies have examined the active molecular mechanisms during cancer progression and chemoresistance development. The discovery of lncRNAs provides a new entry point for us to study the mechanism of cisplatin resistance. Accumulating evidence has emphasized that lncRNAs possibly function as biomarkers and influence cancer chemoresistance^30,31^. LncRNA UCA1 could increase CDDP resistance and inhibit cell apoptosis in oral squamous cell carcinoma (OSCC)^32^. LncRNA HOXD-AS1 enhanced the sensitivity of glioma to cisplatin by competitively binding to miR-204^33^. GAS5 is a key tumour suppressor gene that has attracted much attention recent years, the content of GAS5 in different types of tumour tissues is lower than that in adjacent normal tissues, and it plays an important role in controlling tumour progression and prognosis, invasion and metastasis, apoptosis and radiotherapy tolerance^34,35^. In colorectal cancer tissue samples, the expression of GAS5 was significantly lower than that of adjacent normal tissues. The tumour volume of the GAS5 low-expression group was large. In addition, patients with low GAS5 expression showed a shorter survival time. Statistical analysis revealed that the expression level of GAS5 was an independent risk factor and prognostic factor for colorectal cancer^36^. Our study analysed clinical specimens and revealed that GAS5 was elevated in CC tissues compared to corresponding normal cervical tissues. Low GAS5 expression was associated with a younger age, vaginal recurrence or metastasis, chemotherapy and poorer OS in CC patients. Which suggested that lncRNA GAS5 may be a potential prognostic indicator of CC associated with chemotherapy.

We further explored the mechanisms of GAS5 on the sensitivity to chemotherapy. STATs, a family of latent cytoplasmic proteins, can transduce signals from cytokine and growth factor receptors to the nucleus. Activated STATs are phosphorylated on tyrosine residues and then dimerize via reciprocal SH2 phosphotyrosine interactions and enter the nucleus to regulate the transcription of many different genes by binding to their promoter regions^37^. Persistent STAT activation in tumour cells abolishes the control of cell growth, survival, angiogenesis and immune function. To date, STAT3 is the most comprehensively explored STAT family member in terms of its contribution to cancer^38^. STAT3 signal is frequently activated during cancer development and associated with different characteristics of cancer. Research found that STAT3 was not activated in the periphery of non-tumour tissue and in the normal liver but was activated in hepatocellular carcinoma^39^. Recent studies verified that abnormal activation of STAT3 signalling mediates cisplatin sensitivity in several cancers, such as breast cancer, OSCC, oesophageal squamous cell carcinoma and ovarian cancer and lung cancer^40–44^. In CC, overexpression of STAT3 was associated with cisplatin sensitivity^45^. Our study discovered that the phosphorylation levels of STAT3 in CC cells HeLa and SiHa were different from those in normal cervical squamous epithelial cells H8. Similarly, P-STAT3 was highly expressed in CC tissues compared to adjacent tissues. What is more, the expression of STAT3 in CC tissues was negatively correlated with the expression of GAS5. Therefore, we speculated that the role of GAS5 in the cisplatin sensitivity of CC was related to activation of STAT3. Via ChIP assay, we found that P-STAT3 could bind to the promoter region of GAS5. Validated that P-STAT3 is a transcription factor of GAS5. Additionally, further functional experiments demonstrated that inhibition of STAT3 phosphorylation promoted the expression of GAS5. Collectively, the above results suggested that P-STAT3 affected the transcription of GAS5 as a transcription factor.

ceRNA is a transcript that can regulate each other at post-transcription level by competing miRNAs^46^. An increasing number of lncRNAs have been identified as ceRNAs that competitively bind with miRNAs to relieve translational repression of targeted mRNAs induced by common miRNAs and their downstream pathways^47^. When ceRNA, such as pseudogenes, is transcriptional silent, parent mRNAs are transcribed and exported to the cytoplasm, which is targeted by microRNA-guided RNA-induced silencing complex (miRNA-RISC) in the cytoplasm, leading to accelerated degradation, blocked translation and decreased expression. When ceRNA acquires transcriptional activity, it competes for miRNA targets, binds to RISC complexes, which results in the separation of miRNA-RISC complexes from parent genes and increased parental gene expression^48^. Miao et al^49^. reported that in ovarian cancer, ANRIL could interact with let-7a to further reduce HMGA2 levels, which promoted the apoptosis and improved the cisplatin sensitivity of ovarian cancer cells. In gastric cancer, lncRNA MT1JP regulated the expression of FBXW7 through competitively binding with miR-92a-3p, and played a role in inhibiting cell proliferation, migration, invasion and promoting cell apoptosis^50^. MiR-21 sponged by GAS5 has been reported in OSCC, hepatocellular carcinoma, laryngeal squamous cell carcinoma and ovarian cancer^13,51–53^. In our study, we verified the same mechanism in CC cells. QPCR analysis after transfection of the miR-21 mimic into cells with stable GAS5 overexpression revealed a negative dual regulatory relationship between GAS5 and miR-21. Furthermore, the results of the dual-luciferase reporter assay revealed that GAS5 contained the binding site with miR-21. RIP and RNA pull-down assays suggested that GAS5 formed an RISC complex with miR-21. Therefore, GAS5 inhibited the expression of miR-21 through “miRNA sponge”.

PDCD4, which is originally found to participate in apoptosis, can selectively enhance chemosensitivity to platinum compounds mainly through the death receptor-mediated pathway^54^. Usually downregulated in tumours^55–57^, PDCD4 can inhibit neoplastic transformation, control translation and induce apoptosis^58^. PDCD4 overexpression activated caspases 8, 9, 3, lead to the apoptosis of liver cancer cells^59^. Our research identified PDCD4 as a target gene of miR-21, which played a key role in apoptosis. PDCD4 expression was higher in CC tissues than in peritumoral tissues. Knockdown of PDCD4 restrained the apoptosis of CC cells. Therefore, we hypothesized that GAS5 promoted cisplatin-induced apoptosis by sponging miR-21 to indirectly regulate the expression of PDCD4. Ultimately, we observed that GAS5 promoted PDCD4 expression both in vitro and in vivo. In contrast, overexpression of miR-21 repressed the promoting effect of GAS5 overexpression on PDCD4 expression and cisplatin-induced apoptosis in CC cells. These data suggested that GAS5 promoted cisplatin sensitivity through the miR-21/PDCD4 axis.

Furthermore, we verified our conclusion in vivo. Research discovered that GAS5 overexpression could decrease the tumour growth, volume as well as weight of nude mice in vivo experiment^60^. Our study found that with cisplatin injection, the tumour volumes in the GAS5 overexpression group presented a declining trend, which was not due to the influence of proliferation but rather apoptosis, indicating that GAS5 overexpression enhanced the cisplatin sensitivity of xenograft tumours. Collectively, these findings indicate that GAS5 is a positive regulator of cisplatin sensitivity in CC, and that targeting GAS5 may be an effective method for combating CC chemoresistance.

## Conclusions

**Fig. 8:**
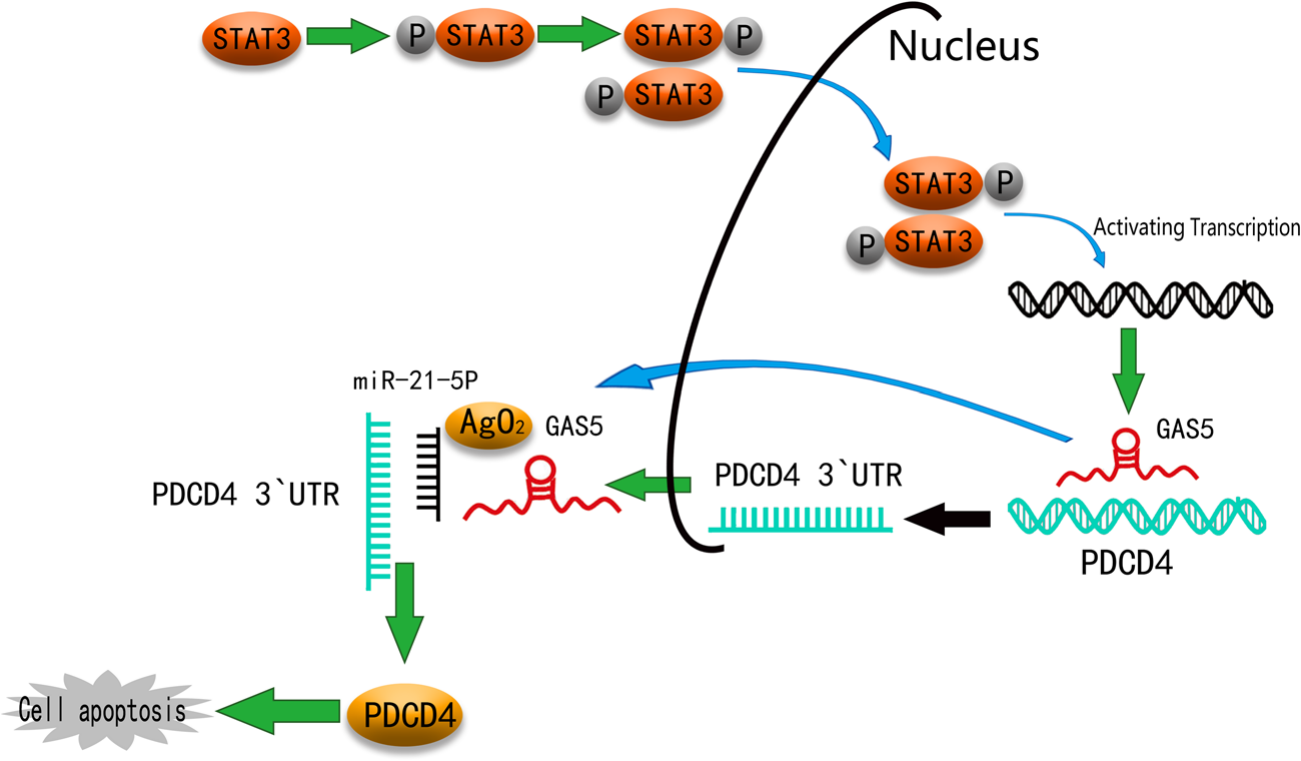
Schematic diagram. GAS5, regulated by P-STAT3, enhances cisplatin sensitivity by acting as a ceRNA to regulate PDCD4 expression in CC.

Overall, our data provided evidence that low GAS5 expression in CC tissues compared to normal tissues was connected with prognosis. Regulated by P-STAT3, GAS5 enhanced cisplatin sensitivity through the GAS5/miR-21/PDCD4 pathway (Fig. 8). Because low GAS5 expression is more likely to occur in young women with CC, therapeutics that target GAS5 may be a promising cisplatin-based chemotherapeutic strategy for use in younger patients.

### Web links and URLs

GEPIA (http://gepia.cancer-pku.cn/)

UCSC (http://genome.ucsc.edu/)

UCSC Xena (https://xenabrowser.net/heatmap/)

JASPAR CORE database (http://jaspar.genereg.net/)

ViennaRNA Web Services (http://rna.tbi.univie.ac.at/)

miRDB (http://mirdb.org/)

GEDS (http://bioinfo.life.hust.edu.cn/web/GEDS/)

## Supplementary information

Supplementary Figure Legends

supplementary figure1

Supplementary figure2

Supplementary table 1

Supplementary table2

Supplementary table3

Supplementary table 4

## Data Availability

All data that support the findings of this study are available from the corresponding authors upon reasonable request.
