# Supplementary material for: Low GAS5 expression may predict poor survival and cisplatin resistance in cervical cancer
Source: Cell Death Dis. 2020 Jul 13;11(7):531. doi: 10.1038/s41419-020-2735-2 (PMC7359315; doi:10.1038/s41419-020-2735-2)
Supplement: Supplementary file 1 — Supplementary Figure Legends [file 41419_2020_2735_MOESM1_ESM.docx]

Supplementary figure1: The relationship between GAS5 expression and survival in the TCGA dataset.

Supplementary figure2: The structure of the psiCHECK2 vector.
