# Supplementary figures and images for: Low GAS5 expression may predict poor survival and cisplatin resistance in cervical cancer
Source: Cell Death Dis. 2020 Jul 13;11(7):531. doi: 10.1038/s41419-020-2735-2 (PMC7359315; doi:10.1038/s41419-020-2735-2)

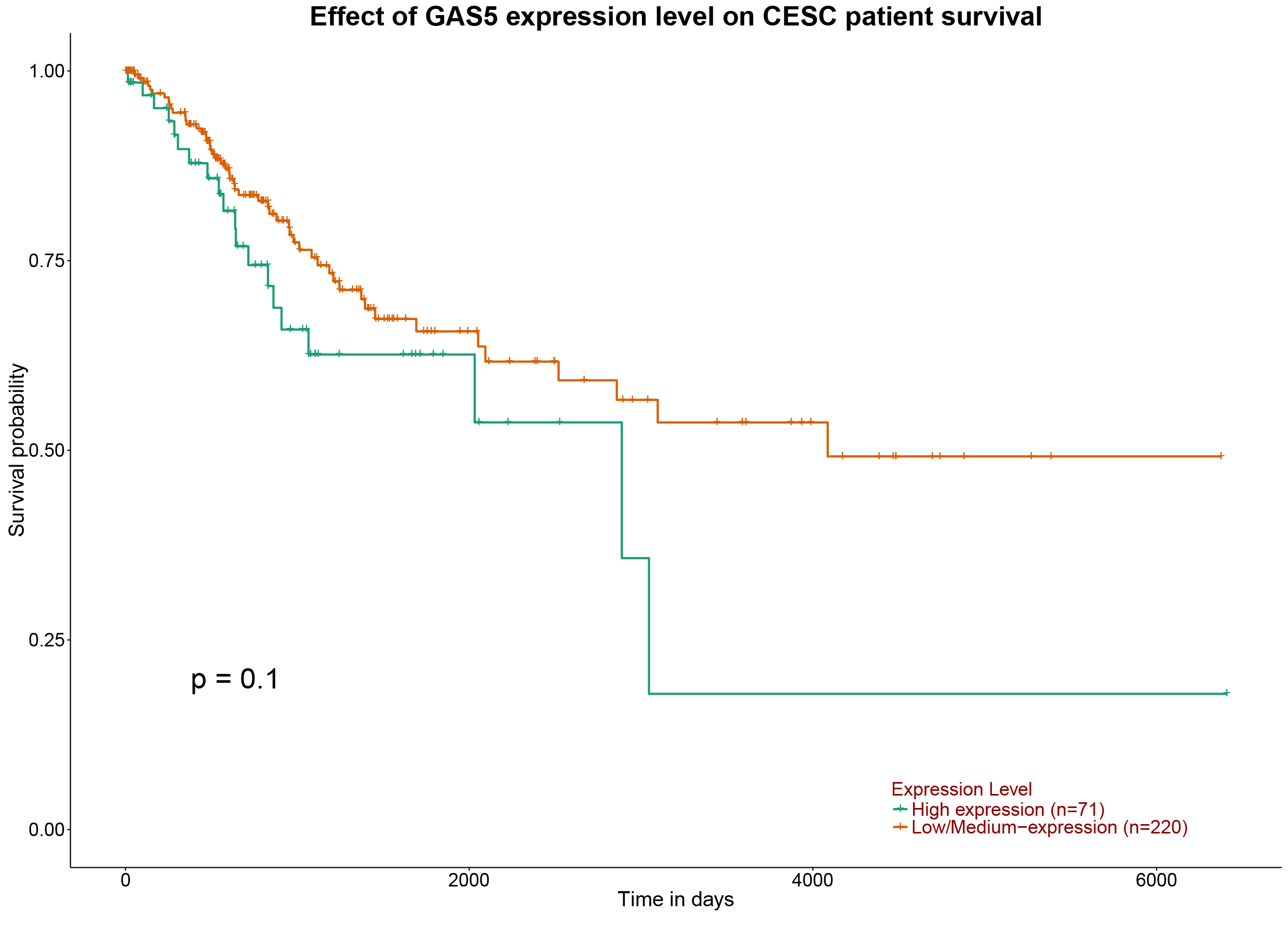

Supplement: Supplementary file 2 — supplementary figure1 [file 41419_2020_2735_MOESM2_ESM.png]

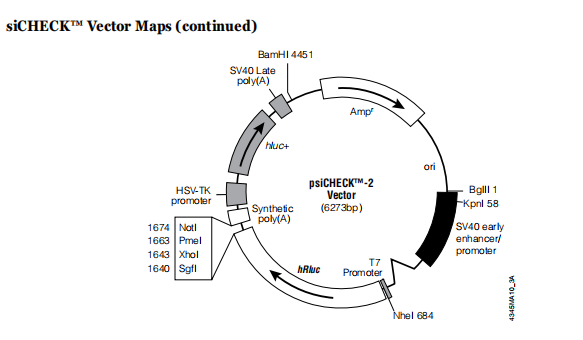

Supplement: Supplementary file 3 — Supplementary figure2 [file 41419_2020_2735_MOESM3_ESM.png]
