# Supplementary material for: Low GAS5 expression may predict poor survival and cisplatin resistance in cervical cancer
Source: Cell Death Dis. 2020 Jul 13;11(7):531. doi: 10.1038/s41419-020-2735-2 (PMC7359315; doi:10.1038/s41419-020-2735-2)
Supplement: Supplementary file 4 — Supplementary table 1 [file 41419_2020_2735_MOESM4_ESM.docx]

Supplementary table 1: QPCR and RT primers

| Primer Name | Sequence |
| --- | --- |
| GAS5 | 5'-TGAAGTCCTAAAGAGCAAGCC-3' |
|  | 5'-ACCAGGAGCAGAACCATTAAG-3' |
| β-actin | 5’-TCGTGCGTGACATTAAGGAG-3’ |
|  | 5’-GTCAGGCAGCTCGTAGCTCT-3’ |
| Uni-Reverse | 5’-GTGCAGGGTCCGAGGT-3’ |
| miR-21 F | 5’-GCACTAGCTTATCAGACTGA-3’ |
| U6 | 5’-CTCGCTTCGGCAGCACA-3’ |
|  | 5’-AACGCTTCACGAATTTGCGT-3’ |
| miR-21 RT -primer | 5’-GTCGTATCCAGTGCAGGGTCCGAGGTATTCGCACTGGATACGACTCAACA-3’ |
| U6 RT -primer | 5’-CGCTTCACGAATTTGCGTGTCAT-3’ |
