# Supplementary material for: Low GAS5 expression may predict poor survival and cisplatin resistance in cervical cancer
Source: Cell Death Dis. 2020 Jul 13;11(7):531. doi: 10.1038/s41419-020-2735-2 (PMC7359315; doi:10.1038/s41419-020-2735-2)
Supplement: Supplementary file 5 — Supplementary table2 [file 41419_2020_2735_MOESM5_ESM.docx]

Supplementary table2: The primer sequences used for ChIP-qPCR

| Primer Name | Sequence |
| --- | --- |
| Binding region 1 | 5′-ACTTTCCTCCAGGGGCTTC-3′ |
|  | 5′-CCTCTGTCGATGCCATCAT-3′ |
| Binding region 2 | 5′- TTTCTTTTATCTGGAGATTTTGAAAC -3′ |
|  | 5′- CTCCATGGTCGTGCCTGAG -3′ |
| Binding region 3 | 5′-CAGCTTCAGGTTTAGTGTTTCA -3′ |
|  | 5′-GAAGTTGGCAATTCCGAGGT-3′ |
| Binding region 4 | 5′- CATCACCACCTCGGAATTG -3′ |
|  | 5′- CCCCACAAAAAGACTTTGGTT -3′. |
|  |  |
