# Supplementary material for: Low GAS5 expression may predict poor survival and cisplatin resistance in cervical cancer
Source: Cell Death Dis. 2020 Jul 13;11(7):531. doi: 10.1038/s41419-020-2735-2 (PMC7359315; doi:10.1038/s41419-020-2735-2)
Supplement: Supplementary file 6 — Supplementary table3 [file 41419_2020_2735_MOESM6_ESM.docx]

Supplementary table3: Logistic analysis in CC tissues

| Comparison between group | OR^[[1]](#footnote-1)^ | 95%CI^[[2]](#footnote-2)^ | *P* value |
| --- | --- | --- | --- |
| GAS5+recurrence or metastasis | 0.316 | 0.130-0.772 | 0.011 |

1. OR: odds ratio [↑](#footnote-ref-1)
2. CI: confidence interval [↑](#footnote-ref-2)
