# Supplementary material for: Low GAS5 expression may predict poor survival and cisplatin resistance in cervical cancer
Source: Cell Death Dis. 2020 Jul 13;11(7):531. doi: 10.1038/s41419-020-2735-2 (PMC7359315; doi:10.1038/s41419-020-2735-2)
Supplement: Supplementary file 7 — Supplementary table 4 [file 41419_2020_2735_MOESM7_ESM.rtf]

Supplementary table 4: Spearman's rank correlation analysis of GAS5 with miR-21 and PDCD4 in TCGA database 
Correlations between GAS5 and miR-21 expression of TCGA database	
	GAS5	mIR21	
Spearman's rho	GAS5	Correlation Coefficient	1.000	-.144*	
		Sig. (2-tailed)	.	.011	
		N	308	308	
	MIR21	Correlation Coefficient	-.144*	1.000	
		Sig. (2-tailed)	.011	.	
		N	308	308	

*. Correlation is significant at the 0.05 level (2-tailed).	

Correlations between GAS5 and PDCD4 expression of TCGA database	
	GAS5	PDCD4	
Spearman's rho	GAS5	Correlation Coefficient	1.000	.226**	
		Sig. (2-tailed)	.	.000	
		N	308	308	
	PDCD4	Correlation Coefficient	.226**	1.000	
		Sig. (2-tailed)	.000	.	
		N	308	308	

**. Correlation is significant at the 0.01 level (2-tailed).	
